# Double Lateral Sliding Bridge Flap versus Laterally Closed Tunnel for the Treatment of Single Recessions in the Mandibular Anterior Teeth: A Pseudorandomized Clinical Trial

**DOI:** 10.3390/jcm11102918

**Published:** 2022-05-22

**Authors:** Norberto Quispe-López, Juan Sánchez-Santos, Joaquín Delgado-Gregori, Joaquín López-Malla Matute, Nansi López-Valverde, Álvaro Zubizarreta-Macho, Javier Flores-Fraile, Cristina Gómez-Polo, Javier Montero

**Affiliations:** 1 Department of Surgery, Faculty of Medicine, Dental Clinic, University of Salamanca, Miguel de Unamuno Campus, 37007 Salamanca, Spain; alvaro.zubizarreta@usal.es (Á.Z.-M.); j.flores@usal.es (J.F.-F.); crisgodent@usal.es (C.G.-P.); javimont@usal.es (J.M.); 2 Department of Surgery, Salamanca Biomedical Research Institute (IBSAL), University of Salamanca, 37007 Salamanca, Spain; nlovalher@usal.es; 3 Department of Surgery, Implants and Periodontics, Faculty of Health Sciences, Alfonso X El Sabio University, 28691 Madrid, Spain; odnsanchez_@hotmail.com (J.S.-S.); jdelggre@uax.es (J.D.-G.); jlpezmat@uax.es (J.L.-M.M.)

**Keywords:** root coverage, esthetics, mandibular anterior teeth, connective tissue graft, laterally closed tunnel, double lateral sliding bridge flap, single gingival recessions

## Abstract

(1) Background: This study compared the clinical and esthetic results of the double lateral sliding bridge flap (DLSBF) and the laterally closed tunnel (LCT) techniques, with a subepithelial connective tissue graft (SCTG), for the treatment of single Miller class II-III recessions in the mandibular anterior teeth. (2) Methods: This pseudorandomized clinical trial evaluated 14 patients, 7 of whom were part of the DLSBF + SCTG group, with an average follow-up of 58.7 ± 24.0 months, and 7 of whom were in the LCT + SCTG group, with an average follow-up of 16.7 ± 3.3 months. Clinical and esthetic evaluations of the following parameters were performed and the results for the two groups were compared: gingival recession depth, probing depth, keratinized tissue width, gingival thickness, percentage of root coverage and root coverage esthetic score. (3) Results: After the follow-up period, each technique provided evidence of a reduction in recession depth and clinical attachment level, as well as increased keratinized tissue width and gingival thickness, with statistically significant differences (*p* < 0.05). The analysis showed that gingival recession depth decreased less in the DLSBF group (4.3 ± 1.2 mm to 0.6 ± 1.1 mm) than it did in the LCT group (4.9 ± 1.1 mm to 0.1 ± 0.4 mm), but no significant difference was found between the two groups. Similarly, a greater reduction in the clinical attachment level parameter was observed in the LCT group, while a greater increase in gingival thickness was observed in the DLSBF group. The presence of scars was the only parameter for which statistically significant differences (*p* < 0.05) between the two study groups were found. (4) Conclusions: Within the limitations of the study, it indicates that the LCT + SCTG technique may be considered an optimal technique in terms of reducing gingival recession depth, complete root coverage and esthetic results for the treatment of single gingival recessions in the mandibular anterior teeth.

## 1. Introduction

Gingival recession (GR) is defined as the apical shift of the gingival margin with respect to the cementoenamel junction; it is associated with attachment loss and with exposure of the root surface to the oral environment [1]. Its prevalence is close to 60% in individuals ≥30 years of age, while 50% of people between 18 and 64 years have one or more sites with gingival recession [1,2].

There are anatomical and mucogingival conditions around the mandibular anterior teeth that predispose them to the development of gingival recessions. One of the most important factors is the presence of a bone dehiscence, and the mandibular anterior region is primarily characterized by its thin buccal cortical bone [3]. The increasing demand for orthodontic therapies that lead to buccal displacement of the mandibular incisor roots, associated with the thin buccal cortical bone, facilitates the appearance of gingival recessions [4]. Additionally, a recent systematic review reported unexpected dental movements after orthodontic treatment has finished—caused by the wire retainers and resulting in dental and periodontal complications—termed “wire syndrome” [5]. Clinicians may also encounter aberrant frenula with a very coronal attachment, high muscle attachments, a larger avascular surface area, tooth malposition, shallow vestibules and/or thin gingival phenotype [1,2,6].

All of these factors predispose patients to the appearance of Miller class II and III gingival recessions in the mandibular anterior region, with the associated clinical challenges. Further, there is a need for less invasive surgical techniques with fewer vertical releasing incisions, which interrupt the vascularization of the flap and increase post-operative morbidity [7].

Few surgical techniques provide good predictability in root coverage for single gingival recessions in the mandibular area [8,9,10,11,12]. The coronally advanced flap (CAF) technique with connective tissue graft is considered the gold-standard treatment for single Miller class I and II gingival recessions [8,13]. However, the last few years have seen the development of surgical techniques with fewer vertical releasing incisions that offer good clinical results [10,12].

The objectives of this pseudorandomized clinical trial were therefore to compare the clinical efficacy and esthetic results of two distinct surgical approaches: the double lateral sliding bridge flap [12,14] and the laterally closed tunnel [10] techniques, combined with a connective tissue graft, for the treatment of single Miller class II and III gingival recessions in the mandibular anterior teeth. 

## 2. Materials and Methods

### 2.1. Subject Selection

A total of 14 adult patients were included in the study (2 men and 5 women in the DLBSF group, and 1 man and 6 women in the LCT group), aged between 22 and 37 years (mean age 29.0 ± 5.3 years for the DLBSF group and 31.1 ± 4.5 years for the LCT group) who presented a single buccal Miller [15] class II or III gingival recession in the mandibular anterior teeth. 

The patients were selected consecutively from referrals to a private periodontal clinic, between September 2014 and January 2020 for the DLBSF + SCTG group, and from January 2020 to November 2020 for the LCT + SCTG group.

None of the patients were smokers and all were systemically healthy, with no signs of periodontal disease, a plaque index of <20% [16] and no restorations or caries in the area to be treated. The minimum recession depth was 3 mm. Medically compromised patients, pregnant women and patients with an unidentifiable cementoenamel junction were excluded.

Selected patients were informed of the aims of the therapy, as well as the importance for participating in the study of compliance with oral hygiene practices and attendance at appointments. Written informed consent was obtained from all the patients. The study design was approved by the Bioethics Committee of the University of Salamanca (no. 483).

### 2.2. Clinical Assessments

The following clinical measurements were taken at baseline and at the final evaluation after surgery. Except for gingival thickness, all clinical measurements were recorded using a manual periodontal probe (Colorvue UNC 12, Hu-Friedy, Chicago, IL, USA) and rounded up to the nearest millimeter at the midbuccal aspect of the treated teeth.

- Recession type (RT): the Miller [15] classification was used.
- Gingival recession depth (GRD), measured in millimeters as the distance from the cementoenamel junction to the gingival margin.
- Probing depth (PD), measured in millimeters from the free gingival margin to the most apical part of the gingival sulcus.
- Clinical attachment level (CAL): algebraic sum of the PD and GRD.
- Keratinized tissue width (KTW), measured in millimeters from the free gingival margin to the mucogingival junction (MGJ).
- Gingival thickness (GT), determined 2 mm apical to the new gingival margin using a K#10 endodontic file with rubber stop (transgingival probing) [17].
- The percentage of root coverage (RC) was calculated by the following formula: ([recession at baseline–recession at the final examination]/recession at baseline) × 100.
- Evaluation by an operator (N.Q.) of esthetic outcomes after root coverage using the root coverage esthetic score (RES) system, with reference to intraoral photos. The file with preoperative and postoperative images was provided, together with the data collection record for each clinical case, as per the RES instructions. Five variables were evaluated with the RES system after surgery: the level of the gingival margin, the marginal tissue contour, soft tissue texture, the position of the MGJ and gingival color [18]. The final esthetic score ranged from 0 to 10, the maximum score being 10. The score breakdown was as follows: between 0 and 6 points were allocated to the level of the gingival margin, and the marginal tissue contour, soft tissue texture, mucogingival junction and gingival color parameters received either 0 or 1 point each.

All the surgical procedures and clinical measurements were performed by the same experienced periodontist (N.Q.). These clinical measurements were made immediately before surgery (baseline) and at the final follow-up visit. N.Q. is a calibrated examiner who had previously been trained in these clinical parameters with high concordance levels (intraclass correlation coefficient >0.95) [12].

To evaluate the esthetic treatment outcome using the RES index, photographs were taken with a Canon EOS 700D camera, Canon EF 100 mm f/2.8 L Macro lens (Canon, Tokyo, Japan) and two 60 × 60 cm softboxes with studio flash (Neewer, Shenzhen, China). The patient was positioned lying on the dental chair, completely parallel to the floor, while lateral flashes and the camera were located at 12 o’clock, at a distance of 0.49 m, with an f 20 diaphragm positioning the camera lens perpendicular to the longitudinal axis of the experimental tooth. Intra-examiner reproducibility was determined by measuring PD and GRD in five different patients on two occasions, 48 h apart. Calibration was accepted if 90% of the recordings could be reproduced to within 1 mm. All preoperative and postoperative images from each clinical case were imported and matched on a Keynote 2020 slide for evaluation and presentation. For each photograph, calibration was performed using parameterized digital rulers, inspired by the ABFO (American Board of Forensic Odontology) system.

### 2.3. Surgical Approach

The 14 patients were treated consecutively using either the double lateral sliding bridge flap (DLSBF) [12,14] or the laterally closed tunnel (LCT) [10] technique with a simultaneous connective tissue graft. The group assignations were pseudorandomized, in the sense that the first 7 patients who agreed to participate in the study received the DLSBF + SCTG technique and the following 7 received the LCT + SCTG technique.

All mucogingival surgeries were conducted by the same experienced periodontist (N.Q).

**Figure 1:**
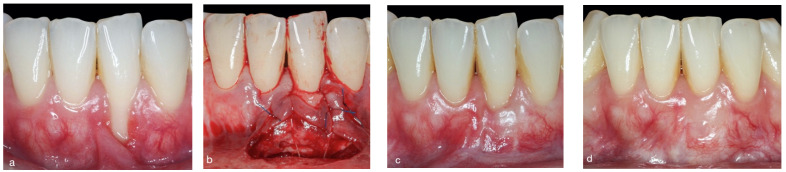
Comparison between baseline (**a**,**d**) and three-year clinical outcome in the DLSBF group. (**b**) In this case, the coronal part of the graft remained exposed. (**c**) After four-month follow-up. (**d**) Final result, having achieved a substantial gain in keratinized tissue width, elimination of frenulum, increase in vestibule depth and absence of apical scar.

**Figure 2:**
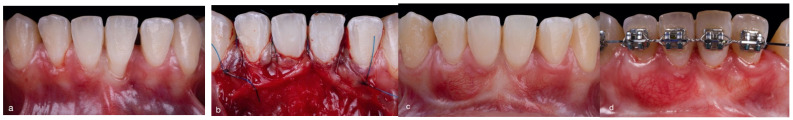
Comparison between baseline (**a**,**d**) and two-year clinical outcome in the DLSBF group. (**b**) In this case, the connective tissue graft was not left exposed but was completely covered by the coronally repositioned flap. (**c**) After four-month follow-up. (**d**) Note the presence of an apical scar after two-year follow-up.

**Double lateral sliding bridge flap plus SCTG group** (Figure 1 and Figure 2): based on a flap design using a single incision at the bottom of the vestibule [12,14]. After local anesthesia, the recipient bed was prepared by making intrasulcular incisions around the affected tooth, using microsurgical scalpels (SB003. Spoon Blade, MJK instruments, Marseille, France). A partial-thickness flap was prepared, extending beyond the mucogingival junction level and leaving the interdental papillae intact. Flap preparation was extended laterally one tooth on either side of the tooth with the recession, using microsurgical scalpels (SB002. Spoon Blade, MJK instruments, Marseille, France). Finally, a single horizontal partial-thickness incision was made into the alveolar mucosa at the bottom of the vestibule. The minimum mesiodistal extension of this horizontal incision was half a tooth on either side of the tooth with a recession, although in certain anatomical situations (aberrant frenula and shallow vestibules) a longer incision may be made, enabling a greater coronal displacement of the flap.

After preparation of the recipient bed, a SCTG of 1.0 to 1.5 mm thickness was harvested from the palatal masticatory mucosa, using the single-incision technique [19]. The donor site was closed with non-absorbable single sutures (5-0 Seralon, Serag-Wiessner, Naila, Germany). The SCTG was then placed into the recipient bed and fixed onto the inner aspect of the flap using two non-absorbable horizontal mattress sutures (6-0 Resotex^®^, RESORBA, Nürnberg, Germany). Additionally, a tooth-suspended suture was inserted, which was anchored in the periosteum and suspended on the lingual aspect of the tooth with a recession, using absorbable 5-0 suture (Serafast^®^, Serag-Wiessner, Naila, Germany). Finally, the flap was mobilized coronally to partially cover the SCTG using a suspension suture (6-0 Resotex^®^, RESORBA, Nürnberg, Germany).

**Figure 3:**
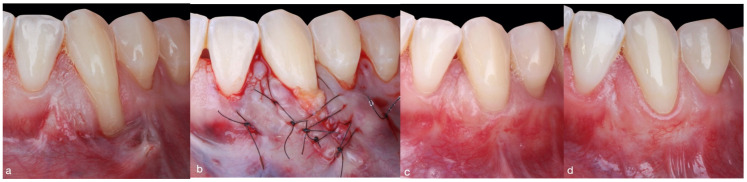
Comparison between baseline (**a**,**d**) and one-year clinical outcome in the LCT group. (**b**) Suturing of the tunnel margins by means of single sutures (6-0 Resotex^®^, RESORBA). (**c**) After four-month follow-up. (**d**) Clinical evaluation at 13 months, at which complete root coverage and a pleasing esthetic appearance have been achieved.

**Figure 4:**
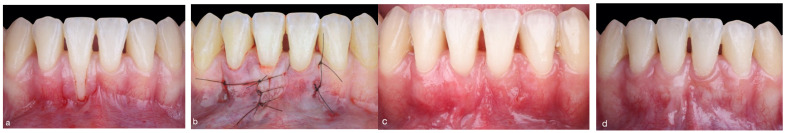
Comparison between baseline (**a**,**d**) and one-year clinical outcome in the LCT group. (**b**) Subepithelial connective tissue graft placed in the tunnel. The tunneled flap was closed laterally by sling sutures and single sutures. (**c**) After four-month follow-up. (**d**) The clinical evaluation at 12 months demonstrates complete root coverage.

**Laterally closed tunnel plus SCTG group** (Figure 3 and Figure 4): This was based on the original LCT [10] design with a palatal subepithelial connective tissue graft, specifically designed for deep single mandibular recessions. After local anesthesia, intrasulcular incisions were made around the tooth with a recession, using microsurgical scalpels (SB003. Spoon Blade, MJK instruments). A tunnel was then prepared, which extended apically beyond the mucogingival junction and mesiodistally one tooth on either side of the tooth with a recession. Muscles and collagen fibers were released from the inner surface of the pouch using microsurgical scalpels (SB003 and SB002, Spoon Blade, MJK instruments) until tension-free displacement of the pouch margins was obtained. As a result of this procedure, the pouch margins could be approximated without tension to partially or completely cover the exposed root surface. 

Subsequently, a palatal SCTG 1 to 1.5 mm in thickness was harvested by means of the single-incision technique [19]. The donor site was closed immediately using single sutures and non-absorbable modified mattress sutures (5-0 Seralon, Serag-Wiessner). The SCTG was then placed into the recipient bed and fixed mesially and distally at the inner aspect of the tunnel using non-absorbable mattress sutures (6-0 Resotex^®^, RESORBA). Next, the graft was adapted to the cementoenamel junction by means of a sling suture. Finally, the margins of the pouch were pulled together over the graft and sutured with interrupted sutures (6-0 Resotex^®^, RESORBA) to accomplish tension-free complete or partial coverage of the graft.

Postsurgically, an Essix-type palatal protection plate was produced for all patients in both groups for greater post-operative comfort. The patients received anti-inflammatory medication (3 × 25 mg/day of dexketoprofen: Enantyun, Menarini, Florence, Italy) for 4 to 5 days, and antibiotics (2 × 1000 mg/day of amoxicillin, Cinfa, Nafarroa, Spain) for 7 days. They were not allowed to brush the surgical sites for 14 days after surgery and were recommended to use a 0.12% + CPC 0.05% chlorhexidine spray (Perio-Aid, Dentaid, Barcelona, Spain) three times a day. The sutures in the donor and recipient sites were removed after 7 and 14 days, respectively. 

Patients resumed tooth brushing 4 weeks after surgery, using the roll technique with a soft toothbrush. Evaluation and photographic monitoring were performed after 1, 3 and 6 months, and at the final follow-up session in November 2021.

### 2.4. Statistical Analysis

Variables reporting quantitative results were expressed as mean ± SD, median, and interquartile range. Variables reporting categorical outcomes were expressed as frequency distributions. Since most of the data did not follow a normal distribution, non-parametric tests such as the Mann–Whitney U Tests were used to evaluate differences between groups for probing depth, gingival recession depth, clinical attachment level, keratinized tissue width, and gingival thickness. Furthermore the pre–post comparisons within therapeutic groups were calculated by Wilcoxon Signed Rank Test. Chi-squared test was also used for compare the distribution of nominal variables between groups.

Statistical analysis was performed using a statistical software program (SPSS Statistics, version 20.0, IBM, Armonk, NY, USA). All tests were considered statistically significant when the *p*-value was <0.05.

## 3. Results

This study included a total of 14 healthy, non-smoking patients (11 women and 3 men) with a single buccal Miller class II or III gingival recession. In one patient, the gingival recession was located in the mandibular left canine, and in the other 13 patients the recessions were located in the mandibular central incisors. Seven cases were treated with the DLSBF technique (3 Miller class II recessions and 4 Miller class III recessions) and seven cases with the LCT technique (1 Miller class II recession and 6 Miller class III recessions). 

The mean age of the two study groups was similar: for the DLSBF group, it was 29.0 ± 5.3 years (ranging from 22 to 36) and for the LCT group, it was 31.1 ± 4.4 years (ranging from 26 to 37). The mean observation time for the DLSBF group was 58.7 ± 24.0 months (ranging from 21.5 to 86.0 months), while it was shorter for the LCT group, at 16.7 ± 3.3 months (ranging from 11.6 to 22.0 months). 

Table 1 shows the changes in the clinical parameters for both surgical techniques from baseline to the final follow-up period, and the comparison of the two groups. Table 2 shows the effect of the type of technique on the presence of scars and on the mean percentage of complete root coverage (CRC) in the two study groups. 

The mean percentage of root coverage was 86.9% ± 28.0% for the DLSBF group, in comparison to 96.4% ± 9.4% for the LCT group, with no statistically significant difference (*p* > 0.05) identified between the two techniques. In the DLSBF group, CRC was achieved in five of the seven cases (71.4%), whereas it was achieved in six of the seven cases (85.7%) in the LCT group, with no statistically significant difference between the two groups. Statistically significant differences were identified (*p* < 0.05) with respect to the appearance of scars: in the LCT group, no scars were found (0/7 cases; 0%), whereas all the patients who presented scars belonged to the DLSBF group (3/7 cases; 42.9%).

At the end of the follow-up period, the GRD, CAL, KTW and GT parameters were statistically significant (*p* < 0.05) in both the DLSBF and LCT groups, after comparison using the non-parametric Wilcoxon signed-rank test. However, upon comparing the groups using the non-parametric Mann–Whitney U test, no significant differences were found for any of the parameters analyzed.

In the DLSBF group, the mean KTW increased from 0.4 ± 0.5 mm to 3.6 ± 1.5 mm on average, corresponding to a mean gain in keratinized tissue of 3.1 ± 1.3 mm. Similarly, the mean KTW increased from 0.3 ± 0.5 mm to 3.7 ± 1.1 mm in the LCT group, resulting in a mean increase in keratinized tissue of 3.4 ± 1.3 mm. There was therefore no significant difference in the mean keratinized tissue gain between the two study groups. 

The mean CAL values underwent an average decrease of 3.3 ± 1.6 mm for the DLSBF group and a decrease of 5.4 ± 1.8 mm for the LCT group. In contrast, there was a mean increase in GT of 1.2 ± 0.2 mm for the DLSBF group and 1.0 ± 0.1 mm for the LCT group, but this difference between the groups was not significant (*p* > 0.05).

Finally, as shown in Table 3, the final RES score for the DLSBF group was 8.1 ± 2.5 out of 10, while it was 9.0 ± 1.8 out of 10 for the LCT group, with no significant difference found between the study groups. Given that all patients had a score of zero on the RES scale at the beginning of the study, these results mean that there was an improvement of 81% in the esthetic dimensions for the DLSBF group and 90% for the LCT group. 

## 4. Discussion

The increasing prevalence of patients with gingival recessions in the mandibular anterior region makes it necessary to evaluate the effectiveness of distinct contemporary surgical techniques that can sufficiently solve this problem. Selecting the most appropriate type of surgical technique for the treatment of single Miller class II and III gingival recessions in the mandibular anterior region is a considerable challenge due to the anatomical conditions of that area [20,21]. Whether one uses the gingival recession classification system of Miller [15] or Cairo et al. [22], it is essential to categorize the size of the defect according to the depth of the recession (shallow: <3 mm; moderate: 3–5 mm, or deep: >5 mm). Clinicians are well aware that the deeper the recession, the lesser the likelihood of achieving complete root coverage [23]. Moreover, in the mandibular anterior region, particularly close attention must be paid to the depth of the vestibule and the presence of aberrant frenula with a very coronal attachment [1]. A second and equally important task is selecting a surgical technique that is easy to reproduce, has low morbidity, and offers satisfactory esthetic results. 

The findings of the present study show that single recessions in the mandibular anterior region may be treated successfully using the DLSBF and LCF surgical approaches with simultaneous use of a connective tissue graft (Table 1). Both the DLSBF and LCT surgical techniques achieved a very similar mean percentage of root coverage. For the DLSBF group, the mean root coverage percentage was 86.9 ± 28.0%, with CRC obtained in 71.4% of the sites treated. In the LCT group, we observed a higher percentage of mean root coverage (96.4 ± 9.4%) and CRC (85.7%), and found no significant difference between the two groups. This is probably due to the limited number of recessions treated in the two groups. In line with these results, case study series that evaluated the effectiveness of the DLSBF plus SCTG technique for Miller class I and II defects located in the mandibular anterior region reported a mean root coverage percentage of 90.6% ± 16.8% and a CRC rate of 73.3% [14]. 

Studies that evaluated the LCT plus SCTG technique in recessions located in the mandibular anterior region reported mean root coverage percentages of 96.11% and 96.09%, and a CRC rate of 70.83% and 50% [10,24]. 

In the present study, statistically significant differences were only observed between the two groups for the presence-of-scars parameter (*p* < 0.05). However, each surgical technique independently delivered a significant average increase in gingival thickness (*p* < 0.05), showing particular improvements in the gingival phenotype with the DLSBF technique when compared to the LCT technique (1.2 mm ± 0.2 mm vs. 1.0 mm ± 0.1 mm). This may be due to the fact that, as well as the use of a SCTG with both techniques, in the DLSBF technique, the SCTG is completely covered (in most cases) by the coronally repositioned flap. In contrast, with the LCT technique, the most coronal part of the SCTG generally remains exposed, which may be one of the main explanations for the smaller increase in thickness observed at the end of the follow-up period. An improvement in GT not only prevents recurrence of the gingival recession, but it also helps clinicians to select the appropriate treatment approach when they encounter thin phenotypes [1,25]. In our study, greater reductions in CAL were observed with the LCT surgical technique than with the DLSBF surgical technique (5.4 mm ± 1.8 mm vs. 3.3 mm ± 1.6 mm), and these differences were statistically significant (*p* < 0.05). This favorable result for both mucogingival surgery techniques demonstrates the improvement achieved with respect to the baseline CAL (Table 1). This significant reduction for the LCT group may result from the fact that the baseline PD values for the group were greater than the PD values for the DLSBF group (3.7 mm ± 2.1 mm vs. 2.3 mm ± 0.8 mm), as well as the fact that the LCT group included six Miller class III recessions, in comparison to the DLSBF group, which had four Miller class III recessions. Therefore, greater PD values were presumably found in the LCT group due primarily to the presence of deep bone dehiscences located in the mandibular anterior teeth [3]. 

One of the main reasons for selecting one type of surgical procedure over another is to obtain satisfactory esthetic results [20,26]. This is why this study examined whether scars were present after a mean follow-up of 58.7 ± 24 months in the DLSBF group and 16.7 ± 3.3 months in the LCT group. In the LCT group, no scars were found (0/7 cases; 0%), while all the patients who presented scars belonged to the DLSBF group (3/7 cases; 42.9%), and this difference between the two groups was significant (Table 2). It would therefore be recommendable to select the LCT technique when superior esthetic results are required. In line with these results, several studies have confirmed the presence of scars associated with the DLSBF technique in over 50% of cases [12,14]. 

Regarding the gold-standard surgical technique (coronally advanced flap + connective tissue graft), a recent study [9], in which 20 patients with single Cairo class RT1 recessions (Miller class I and II) affecting the mandibular incisors were treated, resulted in a mean root coverage percentage of 98.3% ± 5.2% and CRC in 90% of the cases. After one year, significant changes were observed in the clinical parameters of recession depth, clinical attachment level, keratinized tissue height and gingival thickness. The main disadvantage reported was the mean gain in keratinized tissue height, with a mean gain of 1.05 ± 1.4 mm achieved using the CAF + CTG technique. This increase was much smaller than the mean gains obtained in our study (3.1 ± 1.3 mm for the DLSBF group and 3.4 ± 1.3 mm for the LCT group). This may be considered a positive result for the two techniques evaluated in our study in comparison to the CAF + CTG technique, primarily because of the importance of having adequate keratinized tissue height around our teeth. Additionally, from an esthetic point of view, the color of the coronally repositioned alveolar mucosa would not match the color of the keratinized gums of the adjacent teeth, with a negative effect on the esthetic assessment. 

With regard to the esthetic effect, high RES scores [18] were obtained in both groups (8.1 ± 2.5 for the DLSBF group and 9.0 ± 1.8 for the LCT group) although no significant difference between them was observed. A systematic review with meta-analysis, conducted by Cairo et al. in 2020 [27], evaluated the effect of different flap designs and graft materials for the treatment of gingival recessions, using the RES scoring system. One of its most notable results was that the use of a SCTG is significantly associated with a higher RES score, and the mean esthetic scores achieved with techniques using a SCTG ranged between 6.4 ± 3.7 and 9.3 ± 1.1. However, Cairo et al. [27] only analyzed the coronally advanced flap and tunnel techniques, which are distinct to those used in this study.

The rationale for separately analyzing whether scars were present in our study was that 60% of the points in the RES scoring system are given to the level of the gingival margin, whereas the presence of scars, mucogingival junction alignment, gingival color and marginal tissue contour, which are very important parameters for evaluating esthetic results, are assigned quite a low value (10%). It may be desirable to give equal weight to the various parameters of the index, given that the esthetic impact (scars, color, texture, etc.) of the other parameters might be considered even more important by patients than the gingival margin. It would be desirable for future studies to evaluate the specific weight of each parameter in patients’ therapeutic satisfaction.

## 5. Conclusions

While bearing in mind the limited number of cases in each study group, the following conclusions may be drawn.

The LCT plus SCTG technique may be successfully applied in the treatment of moderate and deep single gingival recessions affecting the mandibular anterior teeth when superior esthetic results are required.

More frequent occurrence of scars was observed in the DLSBF group, and these differences between the two study groups were statistically significant (*p* < 0.05).

LCT plus SCTG tends to produce a better final RES than DLSBF plus SCTG, but this difference between the groups was not significant.

## Figures and Tables

**Table 1:** Comparison of the clinical parameters measured before and after the surgery in the 14 patients treated (7 with the DLSBF + SCTG technique and 7 with the LCT + SCTG technique). Nonparametric test., i.e., Mann–Whitney and Wilcoxon tests were used for inter and intragroup comparisons, respectively.

	All Patients (*n* = 14; 100%)	Double Lateral Sliding Bridge Flap Group (*n* = 7; 50%)		Laterally Closed Tunnel Group (*n* = 7; 50%)		Comparison	
Parameters	N	Mean ± SD	Range	Mean ± SD	Range	U Mann–Whitney	U Statistics *p*-Value
GRD (mm)							
Baseline	14	4.3 ± 1.2	3–6	4.9 ± 1.1	3–6	2.6	0.11
Final follow-up	14	0.6 ± 1.1	0–3	0.1 ± 0.4	0–1	0.4	0.52
Pre–Post Difference	14	3.7 ± 1.6	1–6	4.7 ± 1.2	3–6	2.6	0.11
Wilcoxon Pre–Post comparison (*p*-value)	0.0 (*p* = 0.018) *			0.0 (*p* = 0.017) *			
PD (mm)							
Baseline	14	2.3 ± 0.8	1–3	3.7 ± 2.1	1–7	0.3	0.59
Final follow-up	14	2.7 ± 1.1	2–5	2.9 ± 0.9	2–4	0.3	0.59
Pre–Post Difference	14	(−0.4) ± 1.1	(−2)–1	0.9 ± 1.7	(−1)–3	1.2	0.28
Wilcoxon Pre–Post comparison (*p*-value)	15.0 (*p* = 0.32)			5.0 (*p* = 0.23)			
CAL (mm)							
Baseline	14	6.6 ± 1.4	5–9	8.4 ± 2.1	6–11	2.6	0.11
Final follow-up	14	3.3 ± 1.9	2–6	3.0 ± 0.8	5–9	0.0	1.0
Pre–Post Difference	14	3.3 ± 1.6	0–5	5.4 ± 1.8	3–8	2.8	0.09
Wilcoxon Pre–Post comparison (*p*-value)	0.0 (*p* = 0.026) *			0.0 (*p* = 0.018) *			
KTW (mm)							
Baseline	14	0.4 ± 0.5	0–1	0.3 ± 0.5	0–1	0.3	0.58
Final follow-up	14	3.6 ± 1.5	1–5	3.7 ± 1.1	2–5	0.3	0.59
Pre–Post Difference	14	3.1 ± 1.3	1–5	3.4 ± 1.3	2–5	0.0	1.0
Wilcoxon Pre-Post comparison (*p*-value)	28.0 (*p* = 0.018) *			28.0 (*p* = 0.017) *			
GT (mm)							
Baseline	14	0.0 ± 0.0	0–0	0.0 ± 0.0	0–0	0.0	1.0
Final follow-up	14	1.2 ± 0.2	1–1.5	1.0 ± 0.1	0.8–1.2	2.8	0.09
Pre–Post Difference	14	1.2 ± 0.2	1–1.5	1.0 ± 0.1	0.8–1.2	1.9	0.16
Wilcoxon Pre–Post comparison (*p*-value)	28.0 (*p* = 0.018) *			21.0 (*p* = 0.027) *			
% RC							
% Final follow-up	14	86.9% ± 28.0%	25–100	96.4% ± 9.4%	75–100	28.0	0.53

*: Wilcoxon tests intragroup significant pre–post differences. GRD: gingival recession depth; PD: probing depth; CAL: clinical attachment level; KTW: keratinized tissue width; GT: gingival thickness; %RC: percentage of root coverage; SD: standard deviation.

**Table 2:** Effect of the type of technique on the presence of scars and on complete root coverage in the study sample (*n* = 14).

	Double Lateral Sliding Bridge Flap Group (*n* = 7; 50%)				Laterally Closed Tunnel Group (*n* = 7; 50%)				Chi-Squared Comparison	
Parameters	NO		YES		NO		YES		X^2^	*p*-Value
	N	%	N	%	N	%	N	%		
Scar	4	57.1	3	42.9	7	100	0	0	3.82	0.04 *
CRC	2	28.6	5	71.4	1	14.3	6	85.7	0.42	0.52

*: Chi-squared tests significant. CRC: Complete root coverage.

**Table 3:** Root coverage esthetic score (RES) after the final follow-up in each group and comparison of the two groups (*n* = 14).

	Double Lateral Sliding Bridge Flap Group (*n* = 7; 50%)	Laterally Closed Tunnel Group (*n* = 7; 50%)	Comparison
RES Parameters	Mean ± SD	Mean ± SD	Mann–Whitney U (*p*-Value)
Gingival Margin level	5.1 ± 1.5	5.6 ± 1.1	28.0 (*p* = 0.53)
Marginal Tissue Contour	0.9 ± 0.4	0.9 ± 0.4	24.5 (1.0)
Soft Tissue Texture	0.4 ± 0.5	0.9 ± 0.4	35.0 (0.11)
Mucogingival Junction	0.9 ± 0.4	0.7 ± 0.5	21.0 (0.53)
Gingival Color	0.9 ± 0.4	1.0 ± 0.0	28.0 (0.32)
Total Score (0–10)	8.1 ± 2.5	9.0 ± 1.8	1.2 (*p* = 0.28)

Mann–Whitney tests were statistically significant. RES (Root coverage Esthetic Score): A deep description of the index is in the Materials and Methods section.

## Data Availability

The datasets analyzed during the current study are available from the corresponding author on reasonable request.

## Abbreviations

DLSBF	Double lateral sliding bridge flap
LCT	Laterally closed tunnel
SCTG	Subepithelial connective tissue graft
GRD	Gingival recession depth
PD	Probing depth
CAL	Clinical attachment level
KTW	Keratinized tissue width
GT	Gingival thickness
MGJ	Mucogingival junction
RC	Root coverage
CRC	Complete root coverage
RES	Root coverage esthetic score
